# Evaluation of infrared thermography, force platform and filmed locomotion score as non-invasive diagnostic methods for acute laminitis in zebu cattle

**DOI:** 10.1371/journal.pone.0235549

**Published:** 2020-07-06

**Authors:** Rejane dos Santos Sousa, Francisco Leonardo Costa de Oliveira, Mailson Rennan Borges Dias, Natalia Sato Minami, Leonardo do Amaral, Antonio Humberto Hamad Minervino, Carolina de Lara Shecaira, Juliana Aparecida Bombardelli, Fernando José Benesi, Enrico Lippi Ortolani

**Affiliations:** 1 Department of Clinical Science, College of Veterinary Medicine and Animal Science, University of Sao Paulo (FMVZ/USP), São Paulo, São Paulo, Brazil; 2 Laboratory of Animal Health, LARSANA, Federal University of Western Pará, UFOPA, Santarém, State of Pará, Brazil; University of Lincoln, UNITED KINGDOM

## Abstract

This study aimed to characterize oligofructose-induced laminitis in zebu cattle and comparatively evaluate four different diagnostic methods for laminitis. A total of 29 rumen-cannulated Nelore heifers, weighing 474.5 ± 58.5 kg were used. Laminitis was experimentally induced by intraruminal administration of 0.765 g/kg oligofructose twice daily for three consecutive days, followed by a single dose of 10.71 g/kg oligofructose on the fourth day. The animals were evaluated before administration of the highest dose of oligofructose (basal) and every six hours for up to 24 hours (6, 12, 18, 24 hours) and thereafter, every 12 hours for up to 72 hours (36, 48, 60, 72 hours) post-induction. The following diagnostic methods were used: hoof pain sensitivity test (hoof-testing), locomotion scoring, hoof infrared thermography, and force platform. Diagnosis of laminitis was confirmed after two positive responses to hoof pressure testing. Using a receiver operator characteristic (ROC) curve, we defined the appropriate cut-off for infrared thermography and force plate as 30 °C and < 24%, respectively. From the 29 heifers, 27 developed laminitis (93.1%) which occurred between 24 h to 72 h in the digits from two limbs, with more frequent sensitivity in the lateral digits. Locomotion analysis detected twenty-eight heifers with laminitis and showed that a greater (P = 0.006) number of animals had lameness in two limbs (n = 13; 56%). Using hoof-testing as gold standard for the diagnosis of laminitis the locomotion score displayed 100% sensitivity, 97% specificity and 98% accuracy; infrared thermography showed 96% sensitivity, 63% specificity, and 75% accuracy whilst force plate had 76% sensitivity, 82% specificity and 79% accuracy. This suggests that, for the diagnosis of laminitis in cattle, pain evaluation is more efficient. Considering the difficult to evaluate pain sensitivity in Nelore animals, filmed locomotion score, infrared thermography and force plate methods can be indicated for non-invasive lameness detection in beef farms.

## Introduction

Laminitis is considered a systemic disease with manifestation in the digits, in which there are vascular and degenerative changes of the laminar chorion, affecting the positioning and mobility of the digital phalanx within the horn case [1,2]. Laminitis is a major foot disease in ruminants, but its pathogenesis is still not fully understood [3]. The most accepted hypothesis suggests that the absorption of endotoxins from antigens originating from dead ruminal bacteria could initiate an inflammatory process in the hoof, through activation of inflammatory mediators and metalloproteinases [4–6].

The laminitis model developed by Thoefner et al.[7], where oligofructose overload is used in cattle, has contributed to a better understanding of the disease.. The main clinical signs include temperature increases in the coronary region, pain, edema, and consequent lameness [3]. The diagnosis of laminitis can be made by identifying lameness, assigning a gait score, clinical examination, and using more recent technologies, such as infrared thermography and plate force [8,9].

The locomotion score is a subjective measure that has been shown to be efficient in the diagnosis of lameness. Since it is a cheap method and does not require direct contact with the animal, it has been used in the evaluation of the degree of lameness of beef herds as a screening method, followed by individual clinical examination when necessary [10].

The experience of the observer, as well as the method of evaluation are important points to be considered when evaluating locomotion. Danscher et al. [11] showed that locomotion scores obtained by live observation, differed from those obtained with filming; the latter had an advantage, because of multiple evaluations of the animals leading to verification of subtle details.

Indirect palpation of the heel with hoof testers (sensitivity examination) has been used successfully in the identification of pain. However, although the efficiency of this test is proven, it is important to emphasize that it is influenced by the force applied to the hoof, moisture, and hoof thickness [11]. This method has proven to be efficient in the identification of oligofructose-overload-associated laminitis [7,11–13].

Infrared thermography and force platforms have been used as new tools in the diagnosis of laminitis. Infrared thermography is a non-invasive evaluation method that produces a pictorial representation of the surface temperature of an object, where the color gradient reflects differences in emitted heat [14]. It has been used for several purposes as a non-invasive animal monitoring method, including as a laminitis diagnostic [9,15]. Heat is one of the cardinal signs of inflammation, and its increase is a consequence of elevated blood flow in the affected area. Therefore, monitoring temperature changes in areas, such as the coronary region can aid in detecting developing inflammation [16].

Another feature that can be explored in the diagnosis of laminitis is the distribution of weight between limbs. Force platforms were developed to evaluate weight distribution because animals with untreated limb lesions tend to divert weight-bearing to the contralateral limb, which may be secondarily affected by lameness [17]. Non-invasive methods to detect lameness were successfully used in other animals species [18] and in dairy cattle [19,20] but as far as we know, there is a lack of scientific information regarding Zebu cattle, a breed that has a difficult management since it is more aggressive than taurine breeds. Although oligofructose-induced laminitis in cattle has been established, a better characterization of the disease and an evaluation of different diagnostic methods, taking into account cost and efficiency, are warranted. Thus, we aimed to evaluate different non-invasive diagnostic methods for the diagnostic of laminitis and provide a more detailed clinical characterization of the oligofructose-induced laminitis in beef cattle.

## Material and methods

This study was approved by the Animal Ethics Commission of the College of Veterinary Medicine and Animal Science from the University of São Paulo (Approved protocol #2545300714). The animals used in this study were bought from local producers and used specifically for this study. After the end of the study, the animals had the ruminal canula removed and then were slaughtered.

### Animals and feed

A total of 29, three year old healthy Nelore heifers, weighing a mean ± standard deviation (SD) of 474.51 ± 58.49 kg, were used. The animals were dewormed and vaccinated against clostridiosis; they underwent surgery for ruminal cannula implantation, which was followed by a 30-day recovery period.

The animals received a basal diet calculated at 2.3% of live weight, composed of 60% dry matter of Coast-cross grass (*Cynodon dactylon*) hay and 40% of concentrate, with 14% of crude protein, offered once daily. The cattle were provided 60 g of commercial mineral supplement daily and had free access to water.

### Study design

We designed a prospective experimental study, in which the animals were initially subjected to induction of acute ruminal lactic acidosis using oligofructose, according to a methodology adapted from Thoefner et al.[7] and described elsewhere [1], with the administration of 15.3 g of oligofructose (Beneo P95, Orafti Active Food Ingredients, Chile) per kg of body weight (BW) per animal. Briefly, each heifer received 1.53 g/kg BW (adaptation doses) of oligofructose per day for three consecutive days followed by one main dose of 10.71 g/kg, totaling the 15.3 g of oligofructose per kg of body weight per animal during four consecutive days. During the administration of the adaptation doses (1.53 g/kg) of oligofructose, it was divided in two doses of 0.765 g/kg administered in the morning and afternoon, totaling 1.53 g/kg per day. Oligofructose was diluted in warm water and administered directly through the rumen cannula. During the entire study, the diet (concentrate and hay) was freely available to the animals.

The animals were evaluated for hoof sensitivity by application of pressure with hoof testers, locomotion scoring, infrared thermography and force platform measurement at the following times: 72, 48, and 24 hours before induction (T-72, T-48, and T-24). The baseline (T0) was considered as the moment immediately prior to administration of the main doses (10.71 g/kg) of oligofructose, after which the animals were evaluated every 6 hours for 24 hours (T6, T12, T18, and T24), and then every 12 hours for an additional two days (T36, T48, T60, and T72) to check for the appearance of laminitis using the above-mentioned diagnostic tools.

### Hoof-Testing and diagnosis of laminitis

The hoof sensitivity test (hoof-testing), also known as pain sensitivity test, was performed in cattle at all time-points. All four front claws were examined. The animals were positioned in a holding pen with headgate and holding chute, the anterior limbs were lifted and pressure applied via the hoof testers over the typical site of solar ulceration (axial sole-bulb junction) and the central part of the dorso-abaxial wall. Slight pressure was applied, just enough to visually appreciate the sole horn yield, and a positive reaction was defined as fasiculation in the triceps muscle and/or an attempt to withdraw the leg. Reaction to hoof-testing was subjectively graded as 0 (without reaction to the pressure exerted) and 1 (presence of reaction), according to Danscher et al.[11]. During hoof-testing, the heifers were examined for other claw disorders and we evaluated the tarsocrural joints of all front limbs. The distention was evaluated using a tape measure. Increased values greater than or equal to 2 cm, when compared with baseline, were considered as distention.

The occurrence of laminitis was confirmed when two positive responses to the hoof sensitivity test were seen, in at least one digit of the animal, at two consecutive time-points. In this study, laminitis was defined as a clinical disease with detectable sensitivity and/or lameness, identified shortly after oligofructose overload.

### Locomotion score

Locomotion scoring was performed at all time points. The animals were filmed walking in an open space with a hard and regular surface (concrete floor). The locomotion score was determined by evaluating for the manifestation of pain sensitivity during ambulation. This evaluation was done for each animal at the initial two time-points. The film was examined by four evaluators, who identified the presence of lameness and was considered positive when the locomotion score was equal to or greater than two, according to Sprecher et al. [21] who scored lameness on a 1–5 scale, where 1 was normal locomotion and 5 was severe lameness.

The first instance of sensitivity was usually considered the time of diagnosis, except if animals presented at a subsequent time with a higher number of sensitive limbs, in which case, this latter time was considered the time of diagnosis. Additionally, we used a filmed locomotion score, which allowed to determine in which limb the lameness was present.

### Infrared thermography

Infrared thermography was only performed on the front limbs. It was performed at baseline in all animals, and after confirmation of a diagnosis of laminitis using hoof testers. The animals were taken to a cattle crush placed into a covered area allowing the assessment to be made in the shade. Residual faeces were removed with a spatula and the anterior limbs were photographed. The camera was pointed at the interdigital space of the evaluated limb at a distance of 1 m, and a photograph was obtained in the palmar-dorsal direction [22].

A Flir Systems^®^ thermographic camera (model T620, Stockholm, Sweden) was used, with a high definition 640×480-pixel infrared detector, capable for capturing temperature differences of up to 0.2 °C. The camera had an accuracy of ± 2 °C, field of view of 15° x 11°, focal length of 41mm, range of -40 to +150 °C and it automatically corrects for environmental thermal differences, to minimize the influences of the external environment. The images were always recorded at 9 am.

**Fig 1:**
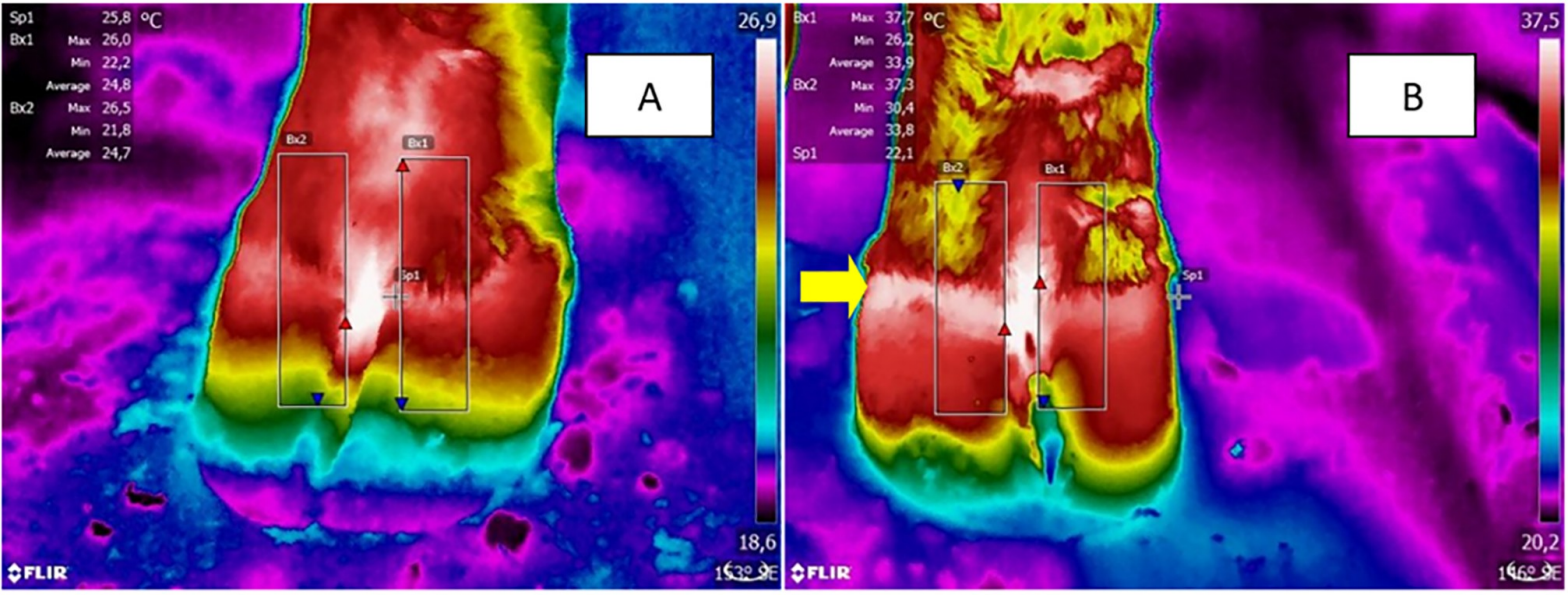
Infrared thermography of the left thoracic limb at baseline (A) and after experimentally induced laminitis (B). The white area indicated by the yellow arrow in the region of the coronary band indicates area of higher temperature.

The images were analyzed using the Flir Systems QuickReport software, with an emissivity of 0.98%, a reflected temperature of 20 °C, a relative humidity of 65%, and an atmospheric temperature of 25 °C. For each photo, a rectangle was designed covering the skin, coronary band, and hoof of each digit, while an average digit temperature was determined. The temperature of each digit was determined by delimiting a rectangle that covered a portion of the hoof, coronary band and skin, and the average temperature of the total area was determined (Fig 1). The cut-off adopted to indicate increased temperature is 27 °C in Europe [23,24]. However, under tropical conditions, this cut-off may not be appropriate, since the experiment was performed in April, when the local environmental temperature was 28 °C. To determine cut-off point for digit temperature, a receiver operating characteristic (ROC) curve test was applied using data from heifers negative and positive for laminitis at hoof-testing (control and patient groups), based on obtained sensitivity and specificity at the confidence interval of 95% (95% CI) using Prisma software (GraphPad Software Inc., San Diego, USA). The area under the ROC curve was 0.85 ± 0.03 (0.80 to 0.90 95% CI; *P* < 0.0001). The cut-off selected was 30.0 °C, which resulted in a 96.2% sensitivity (89.3 to 99.2 95% CI) and 61.0% specificity (51.4 to 70.0 95% CI). For the evaluation of the diagnostic tests, 24 animals with infrared thermography results were used, totaling 96 anterior digits at two points, at baseline and after diagnosis of laminitis by sensitivity tests, which totaled 192 observations.

### Force plate

The assessment using the force plate encompassed all four limbs and was performed at baseline and after confirmation of laminitis. Four force plates were used with independent signal acquisition (50 cm × 30 cm—Signal Acquisition System—EMG System^®^) connected to a computer for data storage, with each platform capable of supporting 250 kg. This equipment was designed and built specifically for the study, following technical recommendations [25,26].

The force plates were installed on a flat surface, and a wooden structure was constructed for leveling the surface of the cattle crush. After the animals entered the cattle crush, all four limbs were positioned on each separate platform for the evaluation of weight distribution. The weight distribution of each limb was recorded at 200 readings/second for 10 seconds. When animals moved such that reading was disturbed, they were repositioned to their original position for a re-read. In the force plate, all the animal limbs were evaluated simultaneously.

The registered weight of each limb was added, accounting for 100%, and then the weight distribution percentage of each limb was calculated. For the evaluation of the diagnostic tests, the weight distributions of 17 animals were used, totaling 34 front limbs, considering the values at baseline and at the moment of initial diagnosis of laminitis, accounting for 68 total observations. Since we had data from hoof-testing and infrared thermography only from the front limbs, we only used the results from the front limbs for the methods comparison. Only 17 animals were evaluated, because of the difficulty of animals adapting to this test.

To determine cut-off point for limb weight distribution, a ROC curve test was applied using data from heifers negative and positive for laminitis at hoof-testing (control and patient groups), based on obtained sensitivity and specificity at the confidence interval of 95% (95% CI) using Prisma software (GraphPad Software Inc., San Diego, USA). The area under the ROC curve was 0.79 ± 0.06 (0.68 to 0.91 95% CI; *P* < 0.0001). The cut-off selected was ≤ 24% which resulted in a 76.5% sensitivity (58.8 to 89.2 95% CI) and 79.4% specificity (62.1 to 91.3 95% CI) (*i*.*e*. 24% or less of weight in one limb, indicating distribution of weight to other limbs due to pain).. We classified the results for each animal, at each time point, as positive or negative [25].

### Statistical analyses

Statistical analyses were performed using Statistical Analysis System (SAS). Data distribution was analyzed by the Kolmogorov-Smirnov test and homogeneity of variance was evaluated. Fisher’s exact test was used to analyze dichotomous variables, sensitivity to pinching, and locomotion score (a score ≥ 2 was considered positive and 1 was considered negative), in order to evaluate the effect among limbs. The Chi-square test was used to evaluate the frequency of laminitis in different digits.

To compare the diagnostic methods of laminitis, the hoof-testing in the anterior limbs was considered as the gold standard. The comparison with locomotion score was evaluated in all 29 animals at two time-points, baseline and after oligofructose induction. The comparison with infrared thermography was evaluated individually per front digit. Both hoof-testing and infrared results were compared from the 4 digits of the 2 front limbs of 24 animals (totaling 192 data points). To compare the force plate results with gold standard, we used data from 17 animals, 2 front limbs per animal at 2 time-points (baseline and after oligofructose induction) accounting to 68 data points. Unfortunately, the infrared thermography and force plate diagnostics methods data were retrieved only from a partial number of animals. This was a technical limitation due to the high amount of management, sampling and data collection at the same time during the experiment.

The presence of true positive results, true negatives, false positives, and false negatives was then assessed using the other diagnostic methods. The following epidemiological indices were calculated: sensitivity, specificity, accuracy, positive likelihood ratio (LR+), and negative likelihood ratio (LR-). The Kappa agreement coefficient was calculated and the strength of agreement was classified as poor (0.00), slight (0.00–0.20), fair (0.21–0.40), moderate (0.41–0.60), substantial (0.61–0.80), almost perfect (0.81–1.00) [27].

## Results

### Hoof-testing

Of the 29 animals used in the study, 27.58% (n = 8) were first sensitive to pain at 24 h, 10.35% (n = 3) at 36 h, 17.25% (n = 5) at 48 h, 20.68% (n = 6) at 72 h, and 17.25% (n = 5) at 60 h; 6.89% (n = 2) were not sensitive to pain.

Of the 27 cattle that were sensitive to pain, 40.75% (n = 11) were sensitive in four digits, 33.33% (n = 9) in three digits, 11.11% (n = 3) in two digits, and 14.81% (n = 4) in a single digit.

When all the hoof-test positive digits (n = 81) were analyzed, there was no difference between the left (n = 40) and right (n = 41) digits (*P*>0.05); however, a higher number of lateral digits (n = 48) than medial digits (n = 33) showed positive sensitivity (*P* = 0.027).

Amongst the 29 study animals, eight (27.6%) presented with an increase in the diameter of the tarsocrural joints, with distension greater than or equal to 2 cm. The animals presented with increased swelling between 24 and 96 h, with a median of 66 h after the induction of laminitis.

### Locomotion score

Of the 29 cattle, 28 were positive at locomotion score, twenty-four animals (83%) had a score of 2, with 3 (10%) animals scoring 3, whilst one animal had a score of 4 (3%). A greater (*P* = 0.006) number of animals presented with lameness in two limbs (n = 13; 56%), when compared to animals with lameness in one (n = 4; 17%), three (n = 5; 22%), or four limbs (n = 1; 4%). The distribution of affected limb combinations in heifers that had two limbs with lameness (n = 13) was also quantified. Most of the animals (n = 6; 46.1%) had lameness in one anterior limb and its ipsilateral correspondent; 23.1% (n = 3) had one anterior limb and the corresponding diagonal limb affected; 15.4% (n = 2) had lameness in two forelimbs and 15.4% (n = 2) in two hindlimbs.

### Infrared thermography

When the average temperature of the hoof was assessed by infrared thermography at baseline, there was no observable difference in the digits of the anterior limbs between the animals (*P* = 0.956). Considering the positive result at the hoof testing as criteria for laminitis definition, we observed an increase in the hoof temperature (*P* = 0.0001) that occurred similarly in all digits of the forelimbs (Table 1).

**Table 1:** Mean, standard deviation and Coefficient of Variation (CV) of the mean temperature (°C) of the anterior and lateral digits of bovines before and after oligofructose-induced laminitis.

Moment	Temperature (°C)—anterior digits				CV
	Right lateral	Right Medial	Left Lateral	Left Medial	
Baseline	27.7±5.4^B^	27.4±4.6 ^B^	28.1±4.3 ^B^	28.1±4.2 ^B^	16.54
Laminitis	33.4±2.8^A^	33.4±2.9 ^A^	33.4±2.9 ^A^	33.2±3.3 ^A^	8.92

Different capital letters in the same column indicate difference between mean temperature the baseline and after the appearance of laminitis through Bonferroni test.

### Force plate

At baseline, there was no difference in weight distribution between the four limbs (*P* = 0.1723), however, 53 ± 5.4% of the weight was distributed in the anterior limbs and 47 ± 5.4% in the posterior limbs (*P* = 0.0264). Onset of laminitis altered the weight distribution between limbs (Table 2). Thus, the affected limbs supported less weight compared with their non-affected contralateral limbs (*P* = 0.0001). The comparison of affected anterior and posterior limbs with their healthy contralateral limbs revealed that the latter supported more weight (*P* = 0.0001). There was no difference between the affected anterior and posterior limbs (19.8 ± 3.2 *versus* 21.6 ± 5.1; *P* = 0.2530) or the unaffected anterior and posterior limbs (28.8 ± 5.7 versus 29.8 ± 4.2; *P* = 0.5830).

**Table 2:** Mean and standard deviation of the weight distribution (%) of the affected limbs and their unaffected contralateral limb after oligofructose-induced laminitis.

Limbs	Condition		*P*
	Unaffected	Affected	
Anterior	28,8±5,7^A^	19,8±3,2^B^	0,0001
Posterior	29,8±4,2^A^	21,6±5,1^B^	0,0001
Overall	28,2±6,4^A^	20,3±4,1^B^	0,0001

Superscript letters on the same line indicate differences between the unaffected and affected members through Bonferroni test.

### Comparison of diagnostic methods

Taking hoof testing as the gold standard test, we compared with alternative diagnostic methods: locomotion score (Table 3), infrared thermography (Table 4), and force plate (Table 5). All cattle positive in the hoof-testing (n = 27) scored 2 or greater in the locomotion test, but one of the two animals negative in the hoof-testing had a locomotion score of 2. At baseline, all animals were negative at both hoof-testing and locomotion score. Thus, locomotion score displayed 100.00% sensitivity, 96.77% specificity, an LR+ of 31, an LR- of zero, and an accuracy of 98%.

**Table 3:** Comparison of the locomotion score test with the gold standard hoof testing for the diagnosis of oligofructose-induced laminitis in cattle.

Diagnostic test ^α^	Hoof-testing +	Hoof testing -	Total
Locomotion score +	27	1	28
Locomotion score -	0	30	30
Total	27	2	58
Sensitivity (95% CI)	1.00 (0.87 to 1.00)		
Specificity (95% CI)	0.97 (0.83 to 0.99)		
LR+	31.0		
LR-	0.0		
Accuracy	0.98		
K	0.96		

^α^ Locomotion score compared with hoof test included 58 data points, 29 animals evaluated at baseline and after oligofructose induction;

^β^ Positive likelihood ratio (LR+); Negative likelihood ratio (LR-); Cohen’s Kappa coefficient of agreement (K).

**Table 4:** Comparison of the infrared thermography with the gold standard hoof testing for the diagnosis of oligofructose-induced laminitis in cattle.

Diagnostic test ^β^	Hoof-testing +	Hoof testing -	Total
Infrared thermography +	76	3	79
Infrared thermography -	45	68	113
Total	121	71	192
Sensitivity (95% CI)	0.63 (0.54 to 0.71)		
Specificity (95% CI)	0.96 (0.88 to 0.99)		
LR+	15.8		
LR-	0.4		
Accuracy	0.75		
K	0.55		

^β^ Infrared thermograph compared with gold standard included 192 data (4 digits per front limb, 2 front limbs per animal and 24 animals. Positive likelihood ratio (LR+); Negative likelihood ratio (LR-); Cohen’s Kappa coefficient of agreement (K).

**Table 5:** Comparison of the force plate with the gold standard hoof testing for the diagnosis of oligofructose-induced laminitis in cattle.

Diagnostic test ^γ^	Hoof-testing +	Hoof testing -	Total
Force plate +	26	6	32
Force plate -	8	28	36
Total	34	34	68
Sensitivity (95% CI)	0.76 (0.59 to 0.89)		
Specificity (95% CI)	0.82 (0.65 to 0.93)		
LR+	4.22		
LR-	0.29		
Accuracy	0.79		
K	0.59		

^γ^ Force plate comparison with gold standard included 68 data (2 front limbs from 17 animals at 2 time-points, baseline and after oligofructose induction). Positive likelihood ratio (LR+); Negative likelihood ratio (LR-); Cohen’s Kappa coefficient of agreement (K).

Considering the cut-off value of 30 °C for thermography of anterior limb digits infrared thermography showed 96% sensitivity, 63% specificity, an LR+ of 15.8, an LR- of 0.4, and an accuracy of 75%.

Considering the cut-off value of 24% for weight distribution in the anterior limbs, as detected by the force plate, this diagnostic method had 76% sensitivity and 82% specificity, an LR+ of 4.22, an LR- of 0.29, and an accuracy of 79%.

## Discussion

We efficiently achieved induction of laminitis in 93.1% of Zebu animals, whereas Thoefner et al.[7] achieved 66.7% and Danscheret al. [11] achieved 100% in taurine cows. Thoefneret al. [7] detected symptoms of laminitis 33 h post-induction, while Danscher et al.[11] did after 30 h. In the present study, the onset of laminitis occurred earlier (up to 24 h) in 27.58% of the cases, with an onset distribution in the remaining cases between 36 to 72 h. The early appearance in our study suggests that zebu females might be more predisposed to laminitis, a hypothesis that requires comparative testing.

The gold standard for the diagnosis of laminitis is the hoof sensitivity test. Although Danscher et al. [11] adopted this test, they also critically evaluated it, defining it as subjective, since it depends on the pressure applied by the operator and the willingness of the animal to cooperate. In our study, despite the use of Nellore cattle, there was no difficulty in carrying out the test on animals well contained in a cattle crush, with positive and negative reactions clearly identified by a single operator performing the test. Thus, the major drawback is the need for a suitable cattle crush, not always available in farms.

According to the classic definition of laminitis associated with rumen lactic acidosis, it is an allergic, inflammatory process with aseptic and systemic characteristics, affecting digits and limbs indiscriminately [2,28–30]. Such a definition implies that being systemic, lactic acidosis-induced laminitis equally affects the eight digits of the four limbs. We further analyzed the frequency of laminitis more deeply. Although the presence or absence of laminitis was determined by evaluating pain sensitivity in the anterior digits, locomotion score was calculated for all limbs. The great majority of animals (84.6%) presented with lameness in the anterior limb, but in 15.4% of the cases, lameness did not involve any anterior limb, and in two cows both posterior limbs were affected.

A positive response to pain in the anterior digits was similar between the right and left side, but it was more prevalent in the lateral digits than the medial. This result contrasts with claims that laminitis in the anterior limbs, mostly affects the medial digits [31,32].

In this study, 27.9% of heifers were affected with polysynovitis, with a median onset of 66 h after induction. Danscher et al. [12] verified, using bacteriological examination, that this polysynovitis has an aseptic feature with variable aspects, presenting with inflammatory cells, fibrin, and a generalized increase of blood leucocytes.

Infrared thermography shows an increase in temperature relative to baseline (*P* = 0.0001) in the affected digits. This increase in local temperature is in line with the pain reaction generated by endotoxin exposure and subsequent inflammation [20,33,34].

The force plate indicated no differences in weight distribution between the four healthy limbs, but revealed that the weight supported by the anterior limbs was greater than by the posterior limbs (*p* = 0.0264). The same was observed in dry dairy cows, with the condition reversed during heavy lactation or advanced gestation [35]. The greater weight supported by the anterior limbs is related to the way the limbs are attached to the thoracic cavity, through ligaments and tendons, with a greater capacity to cushion the impact of weight on the feet, mainly on the plantar cushion, while the posterior limbs are inserted through the coxofemoral joint, a rigid structure incapable of reducing impacts [31].

The anterior limbs affected by laminitis supported lower weights when compared with the unaffected limbs at baseline, which was not observed for affected posterior limbs. This was also described by Neveux et al.[25], who argued that when the anterior limbs are affected, there is a natural transfer of weight to the posterior limbs, but not vice versa.

Weight distribution in the affected limbs of heifers with laminitis was always lower than the respective contralateral limb, either in the anterior or posterior axis, confirming a weight transposition described elsewhere using a piece of similar equipment [25,26].

Using hoof testing as a gold standard, locomotion score achieved the best results in detecting laminitis, since it had a high sensitivity (100%) and specificity (97%), accuracy (98%), and a near-perfect agreement [27] indicated by the kappa statistic (k = 0.96).

Although Danscher et al. [11] used the locomotion score as the gold standard, they question its validity, as it uses subjective criteria and is vulnerable to the idiosyncrasies of the observer. However, they concluded that it remains the best method in practice, emphasizing the need to develop another test that is reliable, economical, objective, and sensitive, for the rapid diagnosis of laminitis.

Two alternative diagnostic methods were tested here: infrared thermography and the force plate. The first challenge to circumvent thermography was establishing a cut-off to discriminate between health and morbidity. Certain European studies adopted 27 °C as a cut-off for all digits [23,24]. However, our mean values, obtained from the digits of healthy heifers, were higher. In contrast, the mean values for affected animals were 33.4 ± 3.0 °C.

Thus, the sensitivity of infrared thermography was high (96.2%), but with a specificity below the expected (60.17%), reducing accuracy (75%) and LR+ (2.41) while increasing LR- (15.83). The presence of many false positives among healthy animals resulted in a low specificity. In order to better understand this, the mean coefficient of variation (CV) was calculated at baseline (27.8 ± 4.6, CV = 16.55%) and for the affected animals (33.35 ± 2.9 CV = 8.69%); the CV for healthy animals was 47.5% higher than that of affected ones, indicating a higher dispersion of the data and causing the higher baseline values to exceed the cutoff line. The kappa index for the two tests was 0.52, and reliability was considered to be average.

Although some studies used the force plate test, none of them proposed a cut-off value [17,25,26]. In order to determine the ideal cut-off point, different base values were used in the calculations. The selected value of 24% was very close to the mean values of healthy animals and sensitive enough to detect situations of mild decreased weight support in injured limbs. The findings of Neveux et al.[25] on weight distribution in cattle with foot injury were also fundamental in the interpretation of the results of this test. The sensitivity (76%) and specificity (82%) obtained were good, resulting in an accuracy of 79%. Some animals were considered false negatives because most injuries affected the posterior limbs without correlating with hoof-testing in the anterior limbs. False positives resulted from a large number of cows leaning on one of the anterior limbs, with a weight distribution slightly lower than 24%. It is possible that a longer reading time, of approximately 1 minute, may give limbs more time to stabilize, decreasing the frequency of false positives and negatives.

The choice of the best method to detect laminitis should be made considering the virtues of each test, as discussed by Danscher et al. [11], *i*.*e*.: sensitivity, objectivity, feasibility, and reliability. The results obtained confirm that among the three methods studied, the best is the locomotion score, which is inexpensive, feasible, and very sensitive, but lacks in objectivity, as discussed previously.

Locomotion score through filming, allowed for a more careful evaluation because of the possibility of repeated reviewing. It is also important to take into account the experience of the observers in reducing the subjectivity of the method [36]. We suggest that filming should be used in veterinary practice for a thorough herd examination.

As concerns about the recommendation of alternative methods are arising, our results clearly indicate that the force plate is preferable over infrared thermography. The best evidence is provided by epidemiological indices: although the specificity of thermography is evidently higher than that of the platform, sensitivity is higher for the latter. As for accuracy, there was no clear difference between the two methods. However, LR+, LR-, and kappa index were better for the force plate.

The force plate, has as an advantage, the precision of its results, with immediate readings at very low cost, allowing further regulations of the frequency and duration of reading. One disadvantage is the small size of plates (50 cm × 30 cm), which makes it difficult to position the animal’s limbs and which occasionally required more than 20 minutes for proper alignment. The force plate could be a valuable diagnostic option in dairy farms, if installed in the milk parlor when cows are standing still and can be evaluated daily to detect early-stage laminitis [8].

Both methods have advantages and disadvantages; thermography is easy to perform, does not require complex animal containment, provides an immediate preliminary result, is slightly less accurate, and is cheaper. A disadvantage is the equipment cost and computer required for more detailed data analysis [24].

## Conclusion

The model for oligofructose-induced laminitis adapted in this study should serve as a standard methodology for studies in zebu breeds, because it showed a greater efficiency inducing laminitis.

Although laminitis is considered a localized inflammatory condition that results from a systemic alteration, the occurrence of sensitivity was not uniform in all digits since the lateral digits were more frequently affected. The locomotion score indicates a pattern of laminitis distribution, with ipsilateral limbs more commonly affected.

Considering the hoof-testing as a gold standard test, the best method of diagnosing laminitis was using the locomotion score, followed by using the force plate, and finally by using infrared thermography. This suggests that, when diagnosing laminitis, pain evaluation is more efficient than measurement of temperature increase in bovine limbs. Considering the difficulty to evaluate pain sensitivity in Nelore animals and the results from specificity and sensitivity, filmed locomotion score, infrared thermography and force plate methods can be indicated for non-invasive lameness detection in beef farms.

## Supporting information

S1 Data (XLSX)
